# Intranasal insulin enhances resting-state functional connectivity in Type 2 Diabetes

**DOI:** 10.1371/journal.pone.0324029

**Published:** 2025-05-20

**Authors:** Zongpai Zhang, Vera Novak, Peter Novak, Christos Mantzoros, Long Ngo, Vasileios Lioutas, Weiying Dai

**Affiliations:** 1 School of Computing, State University of New York at Binghamton, Binghamton, New York, United States of America; 2 Department of Neurology, Beth Israel Deaconess Medical Center, Harvard Medical School, Boston, Massachusetts, United States of America; 3 Department of Neurology, Massachusetts General Brigham Hospital, Harvard Medical School, Boston, Massachusetts, United States of America; 4 Department of Medicine, Beth Israel Deaconess Medical Center and Department of Medicine Boston VA Healthcare System, Harvard Medical School, Boston, Massachusetts, United States of America; 5 Department of Medicine, Beth Israel Deaconess Medical Center and School of Public Health, Harvard Medical School, Boston, Massachusetts, United States of America; University of North Carolina at Chapel Hill, UNITED STATES OF AMERICA

## Abstract

Type 2 diabetes mellitus (T2DM) affects cognition and resting-state functional connectivity (rsFC). Intranasal insulin (INI) has emerged as a potential treatment for T2DM-related cognitive decline.

We aimed to evaluate the effect of INI treatment on rsFC with medio-prefrontal (mPFC) and left/right hippocampus (lHPC/rHPC), and their relationship with the cognition, hemoglobin A1c (HbA1c), and homeostatic model assessment of insulin resistance (HOMA-IR) and walking speed. An MRI sub-study of the MemAID trial was conducted, involving a 24-week treatment with either intranasal insulin or placebo. Blood oxygen level-dependent (BOLD) functional MRI (fMRI) images were acquired on eighteen DM subjects at baseline and eleven DM subjects (eight DM-INI patients and three DM-Placebo) at the end-of-treatment. Compared to DM-Placebo treated subjects, DM-INI patients showed increased mPFC-postcentral rsFC, lHPC-frontal rsFC, lHPC-postcentral rsFC, rHPC-frontal rsFC, and lHPC-mPFC rsFC (p < 0.05). The decreased HOMA-IR, which was observed in the MemAID trial, was associated with increased mPFC-basal ganglia rsFC (p < 0.05). This sub-study provides insights into potential mechanisms of INI effects on rsFC that require validation in a larger trial.

## Introduction

Type 2 diabetes mellitus (T2DM), associated with insulin resistance and microvascular disease, accelerates brain aging [1,2] and increases the risk of cognitive decline [1–6]. Studying the biological features of T2DM is crucial because the increased risk of cognitive impairment is likely due to metabolic and vascular disruptions that affect the brain. Brain magnetic resonance imaging (MRI) allows clinical researchers to noninvasively evaluate brain features at structural, functional, and molecular levels. The MRI-related characteristics can act as objective biomarkers, potentially transforming symptom-based clinical fields, including psychiatry [7]. Multimodal MRI analysis has revealed both overlapping and distinct structural and functional changes in T2DM patients, suggesting a complex neuropathology [8]. Functional MRI (fMRI) is particularly useful for detecting alterations in brain functions, shedding light on the impact of T2DM on brain health. fMRI studies have demonstrated that T2DM is associated with decreased perfusion [9], as well as changes in the activity of cognitive networks and functional connectivity, particularly within the default mode network, limbic system, and cerebellum [10–12]. However, despite the growing evidence linking T2DM to brain alterations, the specific mechanisms underlying these changes and their relationship to cognitive decline remain poorly understood. Insulin resistance affects brain insulin signaling and perfusion and cortical activities in brain regions with high energy demands, such as cognitive-related networks [13]. Insulin resistance may be a common pathway for cognitive decline in aging [14], pre-diabetes [15], T2DM, and Alzheimer’s disease [16,17].

Intranasally administered insulin bypasses the blood-brain barrier and delivers insulin directly into the brain tissue along the olfactory and trigeminal pathways, and rapidly accumulates in the cerebrospinal fluid, suggesting effective delivery to the brain [18]. Therefore, intranasal insulin (INI) delivery is a promising tool for delivering drugs to brain tissue [19]. The acute effect of a single INI dose has been studied previously in young healthy adults, older healthy adults, and older adults with T2DM [20–24]. The acute effect of INI on enhancing brain energy [20], including adenosine triphosphate and phosphocreatine levels in young men, has been reported. A single INI dose (40 IU) increased perfusion in the insular and putamen regions of young men [22] and in the occipital and thalamus regions of older adults [21]. We have demonstrated that a single INI dose (40 IU) acutely improves visuospatial memory and increases perfusion in the insular cortex, and cognitive performance after INI treatment is related to vasoreactivity in older adults with T2DM [23]. We have also reported that a single INI dose enhances hippocampus (HPC) resting-state functional connectivity (rsFC), including HPC-mPFC (medio-prefrontal cortex) rsFC, in older adults with T2DM [24]. HPC and mPFC have well-established roles in cognition, especially in memory encoding and retrieval [25].

Our Memory Advancement with Intranasal Insulin in T2DM (MemAID) trial has shown that long-term (24-week, 40IU) INI treatment improved cognition, walking speed, decreased insulin resistance, and increased brain perfusion in the mPFC in T2DM adults and nondiabetic controls [26]. INI treatment did not affect glycemic control (hemoglobin A1c (HbA1c) and fasting plasma glucose in T2DM participants. However, long-term INI effects on brain rsFC and the relationship of brain rsFC with the outcome variables (cognition, walking speed, and insulin resistance) have not been studied. The present study aims to address the long-term INI effects on HPC/mPFC rsFC and their association with outcome variables by employing fMRI techniques. We employed both a hypothesis-driven approach and an exploratory approach. The hypothesis-driven approach focused on testing a priori hypotheses related to HPC-mPFC rsFC, while the exploratory approach allowed for the identification of unanticipated patterns and relationships. By integrating these complementary methodologies, we aim to provide a comprehensive understanding of INI effects on mPFC/HPC rsFC, balancing the rigor of hypothesis testing with the discovery potential of exploratory analysis. We hypothesized that (1) INI has a long-term effect on increasing HPC/mPFC rsFC of T2DM participants, including HPC-mPFC rsFC, (2) the larger HPC/mPFC rsFC is associated with improved cognition, faster walking speed, and less insulin resistance at baseline, and (3) the long-term HPC/mPFC rsFC increases after treatment are associated with increased cognition, faster walking speed, and reduced insulin resistance. In this MemAID sub-study, we tested the above hypotheses by adopting analysis methods that are more robust to any influence by noises or outliers with a small sample size. This study may help understand the mechanism of long-term INI treatment and its relationship with cognitive performance. Our findings provide a foundation for future large studies to explore and generate hypotheses regarding the long-term effects of INI therapy.

## Methods

### Study population

**Fig 1:**
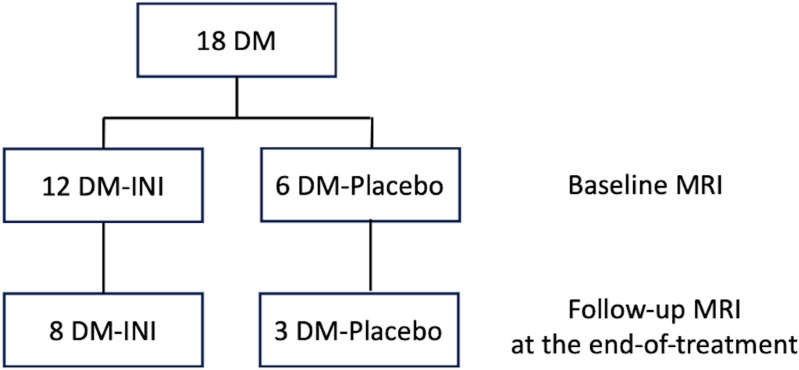
Diagram for the MemAID fMRI sub-study. DM: Type 2 diabetes mellitus; INI: Intranasal insulin. Eighteen participants, twelve DM-INI and six DM-Placebo, completed the baseline MRI scans. Eleven participants, eight DM-INI and three DM-Placebo, completed the end-of-treatment MRI scans. All eleven participants were treatment compliant, using medication daily for more than 109 days (65% of 168 days treatment period). Four DM-INI and three DM-Placebo scans were canceled at the end-of-treatment due to COVID-19.

The MemAID study was a randomized, double-blinded phase 2 clinical trial, consisting of 24-week treatment with 40 IU of human insulin (rDNA origin; Novolin® R, Novo Nordisk Inc., Bagsværd, Denmark) or placebo (0.4 mL bacteriostatic sodium chloride 0.9% solution) intranasally once daily before breakfast and 24-week follow-up period. Novolin® R was used off-label [27]. The trial received approval from the U.S. Food and Drug Administration (FDA: IND#107,690) and was registered on www.clinicaltrials.gov (NCT02415556 as of 4/14/2015). Two hundred twenty-three participants (50–85 years old and able to walk for six minutes), including 106 T2DM and 117 controls, completed the baseline in the MemAID study. The detailed inclusion and exclusion criteria are published elsewhere [26]. In the fMRI sub-study, we aimed to enroll 40 T2DM participants. Participation in the MRI sub-study of the MemAID trial was optional, and as a result, our target enrollment of 40 participants was not achieved in this trial. Eighteen T2DM subjects participated in the fMRI scans at baseline, twelve were assigned with INI treatment (DM-INI) and six were with placebo treatment (DM-Placebo). No statistical differences were observed in baseline demographic characteristics between the 18 T2DM subjects who participated in the sub-study and the 88 T2DM subjects who did not participate (S1 Table). After 24-week of treatment, eight DM-INI and three DM-Placebo subjects participated in the follow-up fMRI scan; seven post-treatment scans were canceled in March 2020 due to the COVID-19 pandemic (Fig 1). Compared to the 7 DM subjects who dropped out at follow-up, the 11 DM subjects who remained had worse verbal memory scores at baseline. No significant differences were observed for other variables (S1 Table). All eleven participants were treatment compliant, using medication daily for more than 109 days (65% of 168-day treatment period). Cognition, gait, and blood samples were obtained in these subjects at baseline and at eight-week periods during 24-week treatment with INI or placebo. The Institutional Review Board of Harvard Medical school approved the study, and all participants fully understood and signed written informed consent. All methods described in this manuscript were performed in strict accordance with the approved guidelines.

### Cognitive and gait measurements and blood sample analyses

Primary assessments encompassed cognitive measurement, normal walking (NW), and dual task walking (DTW) speeds. Verbal memory and executive function were evaluated using the Cambridge Cognition computerized system (CANTAB), which utilized a battery of validated tests [28]. CANTAB was selected because it provides a computerized assessment of cognitive functions and thus reduces an examiner-related assessment bias of tests using the paper versions. Cognitive outcomes were transformed to scaled z scores and combined to form composite cognitive scores [29]. The verbal memory composite score comprised verbal immediate free recall, immediate verbal recognition memory, and delayed verbal recognition memory, where a higher score denotes better performance. The executive function composite score involved paired associates learning (PAL, total errors adjusted) and spatial working memory (SWM, total errors and strategy to complete tasks), with a lower score indicating superior performance.

Participants underwent two six-minutes walking tests on a 45 m indoor hallway. During the first test (NW), they were instructed to walk at their usual and comfortable pace, while during the second test (DTW), they were tasked with performing the same while counting backward (subtracting seven). The time taken to complete each 45 m length and the total distance walked were recorded. No assistive devices were utilized for ambulation, and gait speed was computed by dividing distance (m) by time (s).

Fasting serum glucose, HbA1c, and serum insulin were assessed at Lab Corp (Quest Diagnostics™, Secaucus, NJ, USA). The homeostatic model assessment of insulin resistance (HOMA-IR) was derived by multiplying fasting glucose (md/dl) by insulin levels (mU/L) and dividing the product by 405 [30].

### MRI acquisition

T2DM participants were scanned on a 3 Tesla GE MR750 scanner (General Electric, Milwaukee, United States). The MRI protocol included a localizer, 3D T1-weighted brain volume (BRAVO) images, pseudo-continuous arterial spin labeling (PCASL) perfusion images [31], and blood oxygen level-dependent (BOLD) rsfMRI images. PCASL sequence parameters and image analyses were reported in the other publication. BOLD images were acquired with the following parameters: repetition time (TR): 3196 ms, echo time (TE): 24 ms, field of view (FOV): 24 cm, matrix size: 128x128, slice thickness: 2.5 mm, slices: 58, slice spacing: 2.5 mm, and 124 volumes.

### Image processing

#### Normalization of image time series.

For each subject, the BOLD volumes were transformed to the standard Montreal Neurological Institute (MNI) brain space using Statistical Parametric Mapping (SPM12). Specifically, the first 6 time points of the BOLD time series were removed to increase the stability for further analyses, and the rest of the time series were slice-timing and motion-corrected. All subjects were included in the subsequent analyses as their framewise displace remained within the threshold of 1.875mm (equivalent to one voxel size). The T1-weighted images were segmented into grey matter maps. The grey matter map from each subject was co-registered to the mean of motion-corrected BOLD volumes, and then the motion-corrected BOLD time series for each subject were spatial normalized to the MNI space by applying the transformation parameters from the co-registered gray matter map to the gray matter probability map in the MNI space. The normalized BOLD image time series were smoothed with a 6x6x6 mm Gaussian kernel. Linear regression was used to regress out rigid-body motion and linear detrend. To correct for global signal differences, global BOLD signals (averaged over the whole brain), were regressed out from the BOLD image time series. The regressed volumes were further processed with a band-pass filter of [0.01, 0.1] Hz to get the cleaned BOLD signal time series.

#### Choice of seed locations.

**Fig 2:**
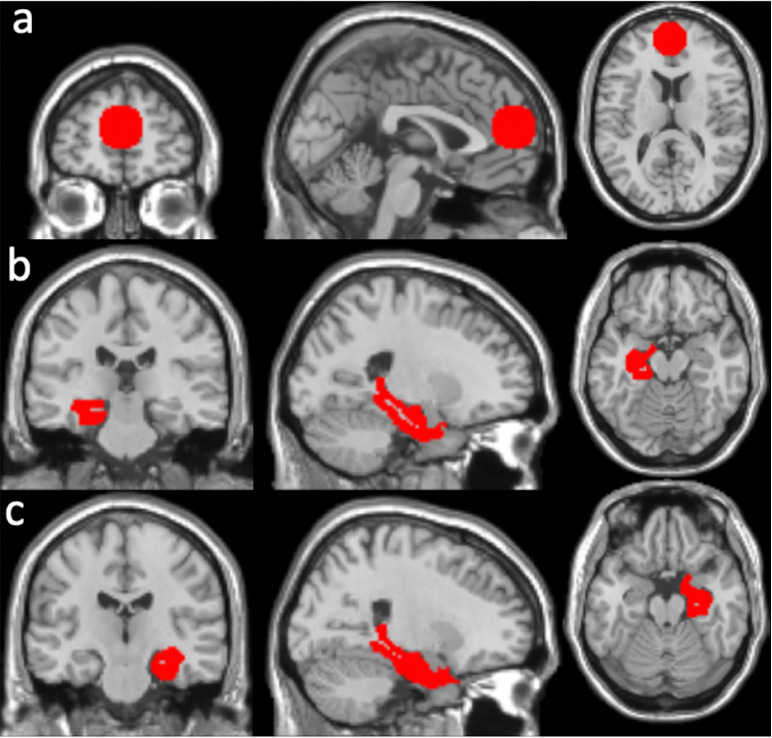
Seed regions of interest: (a) medio-prefrontal cortex, (b) left hippocampus and (c) right hippocampus.

We have previously reported that a single dose of INI acutely increased rsFC between the hippocampus (HPC) and the mPFC cortex, and other default mode network regions in older adults with T2DM as compared with the placebo [24]. Given the observed increase in HPC-mPFC rsFC following the acute INI effect and the key role of the HPC regions in human memory [32,33], we hypothesized that INI has a long-term effect on improving HPC-mPFC rsFC. Additionally, we explored the long-term effects of INI on rsFC by using the mPFC and HPC as seed regions of interest (ROIs). The mPFC seed was selected as the peak location (MNI coordinates [0, 54, 16] mm) in the frontal region, which showed an acute INI effect on HPC rsFC in our prior study [24]. The mPFC seed ROI (Fig 2a) was defined as a sphere with a volume of ~ 4 cm^3^, centered at the seed voxel. The left hippocampus (lHPC) or right hippocampus (rHPC) seed ROIs (Fig 2b and Fig 2c) included the corresponding hippocampus and parahippocampus regions from the automated anatomical labeling (AAL) atlas.

#### Quantification of BOLD rsFC maps.

Individual rsFC maps for each subject were calculated from artifact-cleaned BOLD signal time series using Pearson correlation between time series from each seed ROI and those voxels throughout the whole brain. The time series from each seed ROI was calculated as the mean time series across all the voxels within the seed ROI. For each BOLD signal time series, the rsFC maps from each participant were then transformed into z-score maps by using a Fisher z-transformation to achieve a normal distribution of z-scores at each voxel over the population for group-level statistics.

### Statistical analysis

Due to the small sample size, differences in demographic, neuropsychological, and gait characteristics between the T2DM patients and the controls at either the baseline or the 24^th^-week follow-up and were compared using Mann-Whitney nonparametric U-tests for continuous variables and χ^2^ test for categorical variables; differences between the baseline and 24^th^-week follow-up of T2DM patients were compared using Wilcoxon nonparametric tests.

Due to the potential issues in the data, such as a violation of normality tests in the voxel-based measures and limited sample size, Statistical Non-Parametric Mapping (SnPM, a permutation-based analysis package, http://warwick.ac.uk/snpm) was used to perform voxel-level statistics. SnPM does not assume normal distribution about imaging data [34] and can therefore provide robust results from the limited sample size even in the presence of potential limitations such as non-normality, skewed data, and outliers. In addition, SnPM was shown to be robust with the nominated false positive rate of 5% [35]. Using SnPM, the changes in z-score maps between baseline and end-of-treatment for the eight DM-INI subjects were compared to those of three DM-Placebo subjects using a two-sample t-test model; the z-score maps of eighteen T2DM subjects were associated with outcome variables. No covariates were adjusted due to the relatively small sample size in the voxel-level analysis. The voxel-level significance threshold was set at p < 0.005. The procedure involved generating an empirical distribution of the largest supra-threshold cluster sizes by performing 5000 random permutations. This empirical distribution was used to correct for multiple comparisons among voxels. The cutoff cluster size was determined based on a family-wise error (FWE) rate of 5%, and the significant clusters were identified as those with a corrected p-value of less than 0.05.

Clusters showing significant voxel-level differences between INI-induced and placebo-induced rsFC, as well as those where baseline rsFC values were associated with outcome variables, were defined as ROIs. Based on these ROIs, using linear regression models, we assessed post-hoc differences, baseline associations, and the association between rsFC changes and outcome variable changes. The regional signal value was calculated as the mean signal value over all the voxels within the region. Based on prior literature, T2DM may be influenced by various factors, including age, sex, BMI, diabetes duration, hypertension, waist circumference, and medication use [36–38]. Medication use encompasses subcutaneous insulin, oral and injectable antidiabetic medications, antihypertensive agents, lipid-lowering therapies, and antidepressants. Due to the small sample size, these variables were included one at a time in each basic model to assess their effects as covariates.

## Results

### Basic characteristics of the participants

Table 1 summarizes the participants’ demographic characteristics, cognition, and gait variables. On average, DM-INI participants did not have any significant changes in Body mass index (BMI), insulin, fasting glucose, HOMA-IR, HbA1c, normal walking (NW), dual-task walking (DTW), verbal memory, and executive function after INI treatment. However, three DM-INI participants experienced worsening of their diabetes control, which is unaffected by INI, as indicated by consistently increased levels of plasma insulin (10.60 ± 8.57 uIU/ml), HOMA-IR (3.23 ± 3.05), fasting glucose (11.67 ± 2.08 mg/dl), NW (-9.81 ± 3.96 m/s), and DTW (-10.03 ± 7.79 m/s).

**Table 1:** Summary of demographic characteristics of the diabetes groups.

	Baseline	End of treatment (24-week follow-up)		INI vs. placebo
	DM (n = 18)	DM-INI (n = 8)	DM-Placebo (n = 3)	p value of ΔDM-INI vs. ΔDM-Placebo
Age(years)	64.06 ± 6.97	63.13 ± 5.19	68.67 ± 6.11	N/A
Male/Female	10/8	5/3	2/1	N/A
Hyper/Normo-tension	12/6	5/3	2/1	N/A
Diabetes years	10.44 ± 6.71	8.13 ± 5.62	12.00 ± 3.46	N/A
BMI (kg/m^2^)	30.00 ± 4.26	29.13 ± 2.56	35.27 ± 3.21	0.80
Waist circumference	105.27 ± 10.56	104.50 ± 8.46	105.26 ± 6.04	0.87
Insulin (uIU/ml)	11.89 ± 8.51	13.93 ± 12.49	13.43 ± 2.38	0.83
Fasting Glucose (mg/dl)	149.61 ± 37.68	135.0 ± 19.46	159.67 ± 39.11	0.97
HOMA-IR	4.71 ± 3.69	4.48 ± 4.14	5.22 ± 2.06	0.83
Subcutaneous insulin	0	0	0	N/A
Oral antidiabetic drugs	17	7	3	N/A
Injectable antidiabetic drugs	3	1	0	N/A
Antihypertensive drugs	12	6	2	N/A
Lipid lowering drugs	14	7	2	N/A
Antidepressants	7	2	2	N/A
HbA1c (%)	7.16 ± 1.32	6.71 ± 1.08	6.9 ± 0.26	0.93
NW speeds (cm/s)	112.72 ± 21.08 (n’ = 17)	116.58 ± 12.39	91.38 ± 17.72	0.98
DTW speeds (cm/s)	105.45 ± 34.45 (n’ = 17)	111.94 ± 41.87 (n’ = 7)	85.02 ± 23.52	0.95
Verbal memory z score	-1.15 ± 2.77	-0.72 ± 2.66	-0.38 ± 2.86	0.69
Executive function z score	0.59 ± 2.23	-0.96 ± 2.06	1.18 ± 1.63	0.93

### rsFC Changes from baseline to end-of-treatment

**Fig 3:**
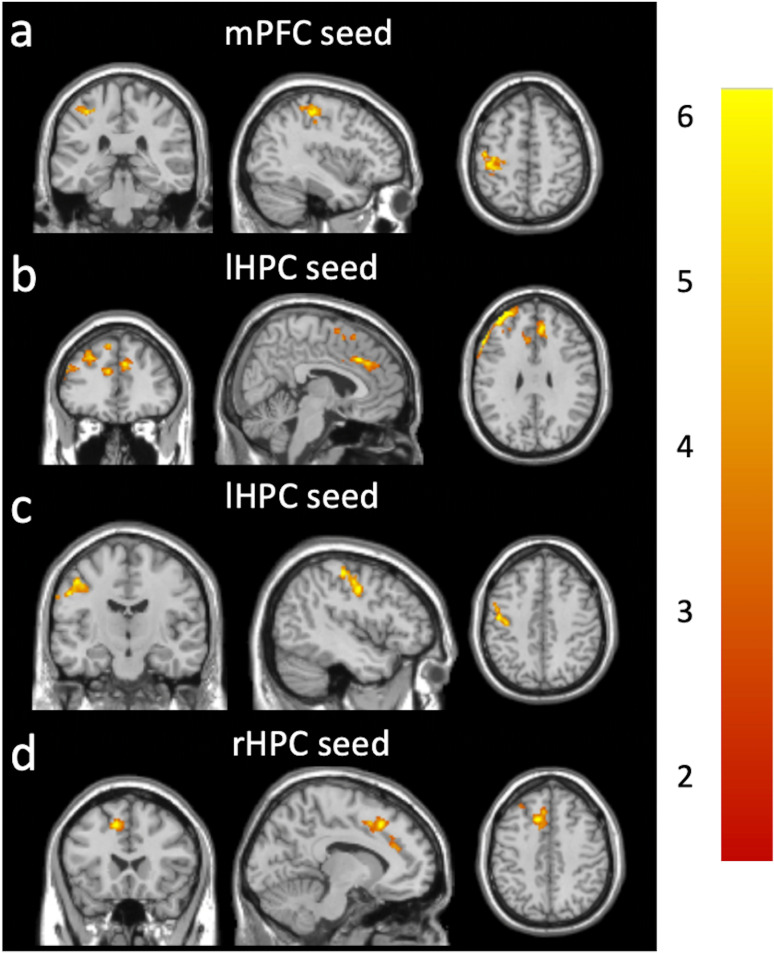
Greater increases in rsFC after 24-week treatment in the DM-INI group (n = 8) compared to the DM-Placebo group (n = 3). The DM-INI group exhibited significantly greater increases in rsFC in the regions: (a) the mPFC with the lPOC, (b) the lHPC with the middle/superior frontal cortex, (c) the HPC rsFC with the lPOC, and (d) the rHPC rsFC with the middle/superior frontal cortex. The color bar shows the range of t-values. mPFC: medio-prefrontal cortex; lPOC: left postcentral; lHPC: left hippocampus; rHPC: right hippocampus.

**Fig 4:**
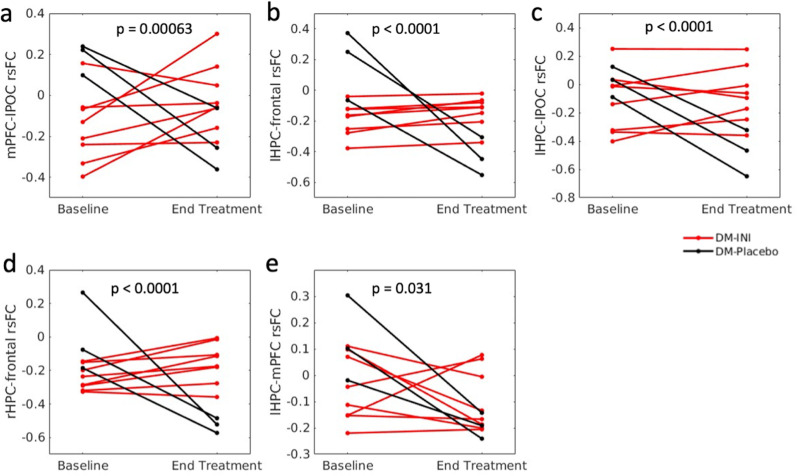
Changes in rsFC from baseline to the end of treatment for DM-INI subjects (n = 8) and DM-Placebo (n = 3) subjects, including post-hoc regional analyses: (a) mPFC-lPOC rsFC, (b) lHPC-frontal rsFC, (c) lHPC-lPOC rsFC, (d) rHPC-frontal and a priori regional analysis: (e) lHPC-mPFC rsFC. P-values indicate significant rsFC differences between the DM-INI and DM-Placebo groups. mPFC: medio-prefrontal cortex; lPOC: left postcentral; lHPC: left hippocampus; rHPC: right hippocampus.

Compared to the rsFC changes in the DM-Placebo group, we observed significantly more increased mPFC rsFC with the left postcentral (lPOC) area (Fig 3a), lHPC rsFC with the middle/superior frontal area (Fig 3b) and the lPOC area (Fig 3c), and rHPC rsFC with the middle/superior frontal region (Fig 3d) in the DM-INI group. Cluster statistics are shown in Table 2. Post-hoc regional analyses showed that DM-INI subjects had larger increases in the mPFC-lPOC rsFC (p = 0.00063, Fig 4a), lHPC-frontal rsFC (p < 0.0001, Fig 4b), lHPC-lPOC rsFC (p < 0.0001, Fig 4c), and rHPC-frontal rsFC (p < 0.0001, Fig 4d). No significant associations were found with any of the investigated covariates (see S2 Table for detailed p-values and partial Pearson correlation coefficients). After adjusting each covariate, the rsFC changes in the DM-INI group remain significant compared to those in the DM-Placebo group. In addition, a priori regional analysis showed that DM-INI subjects experienced smaller decreases in the lHPC-mPFC rsFC (p = 0.031, Fig 4e) than DM-Placebo subjects.

**Table 2:** Summary of cluster-level statistics for clusters showing significant longitudinal changes from the baseline to end-of-INI treatment (n = 8) or significant association with diabetes variables at baseline (n = 18).

	N Voxels	T value of Local	Peak-t MNI coordinates	Anatomical Locations	% Cluster	% Region
Insulin rsFC > placebo rsFC at mPFC (Fig 3a)	340	8.5	-34, -22, 48	**Parietal Lobe**		
				Postcentral_L	82.65	7.22
				**Frontal Lobe**		
				Precentral_L	17.35	1.67
Insulin rsFC > placebo rsFC at lHPC (Fig 3b & 3c)	2903	13.55	-44, 50, 16	**Frontal Lobe**		
				Frontal_Mid_2_L	22.7	14.62
				Frontal_Sup_2_L	19.6	11.68
				Supp_Motor_Area_L Frontal_Sup_Medial_L Frontal_Inf_Tri_L ACC_Sup_L Precentral_L Frontal_Sup_2_R ACC_pre_R Frontal_Sup_Medial_R **Limbic lobe** Cingulate_L	10.61 8.27 7.27 2.86 2.82 2.62 1.31 1.03 7.44	14.35 8.02 8.9 13.72 2.33 1.48 5.86 1.41 9.8
	361	6.55	-50, -16, 38	**Parietal Lobe**		
				Postcentral_L	81.72	7.58
				**Frontal Lobe**		
				Precentral_L	18.28	1.87
Insulin rsFC > placebo rsFC at rHPC rsFC at (Fig 3d)	517	7.58	-32, 58, 20	**Frontal Lobe**		
				Frontal_Mid_2_L	47.58	5.46
				Frontal_Sup_2_L ACC_Sup_L ACC_Pre_L Frontal_Sup_Medial_L ACC_pre_R ACC_Sup_R	28.43 9.48 5.22 3.09 2.71 2.51	3.02 8.10 4.31 0.53 2.16 2.44
	385	6.99	-10, 22, 46	**Frontal Lobe**		
				Frontal_Sup_Medial_L Supp_Motor_Area_L Frontal_Sup_2_L	44.16 23.64 18.44	5.68 4.24 1.46
				**Limbic lobe**		
				Cingulate_Mid_L	13.77	2.73
lHPC rsFC positively	877	6.16	-26, -40, -24	**Cerebellum Lobe**		
				Cerebellum_Crus1_L	42.87	14.44
associated with NW speeds^+^ (Fig 5a)				Cerebellum_6_L Cerebellum_8_L Cerebellum_4_5_L Cerebellum_Crus2_L Vermis_7 Cerebellum_7b_L **Occipital Lobe** Fusiform_L	30.22 9.12 4.79 4.68 1.48 1.14 5.70	15.64 4.24 3.73 2.16 6.70 1.71 2.16
lHPC rsFC positively associated	1564	9.49	-8, -56, -32	**Cerebellum Lobe**		
				Cerebellum_Crus1_L Cerebellum_6_L Cerebellum_Crus1_R	33.50 20.33 10.29	20.13 18.77 6.08
with DTW speeds^+^ (Fig 5b)				Cerebellum_Crus2_L Cerebellum_8_L Vermis_9 Vermis_7 Vermis_8 Cerebellum_Crus2_R Cerebellum_9_L Cerebellum_4_5_L Cerebellum_6_R	5.95 5.44 3.84 3.52 3.45 3.39 3.07 2.81 1.53	4.91 4.50 34.48 28.35 22.22 2.50 5.52 3.91 1.34
				**Occipital Lobe**		
				Fusiform_L	1.21	0.82
rHPC rsFC positively associated with HbA1c^+^ (Fig 5c)	1728	8.73	38, -66, -40	**Cerebellum Lobe**		
				Cerebellum_Crus1_R Cerebellum_Crus2_R Cerebellum_Crus1_L Cerebellum_Crus2_L Cerebellum_6_R Cerebellum_7b_R	50.69 21.01 13.72 7.29 4.63 1.97	33.08 17.15 0.10 6.65 4.46 6.37
mPFC rsFC negatively associated with HOMA_IR^+^ (Fig 5d)	885	6.57	15, 10, 12	**Basal Ganglia**		
				Caudate_R	37.32	34.62
				Caudate_L Putamen_L Putamen_R Pallidum_L	27.82 14.17 1.65 1.08	28.13 14.23 1.63 4.01

* Significant longitudinal changes in rsFC from the baseline to end-of-INI treatment (n = 8) and + significant association with diabetes variables at baseline (n = 18) with a voxel-level p-value threshold of 0.005 and a corrected cluster-level p-value threshold of 0.05 from SnPM.

### Association of baseline rsFC with baseline cognitive variables in T2DM

**Fig 5:**
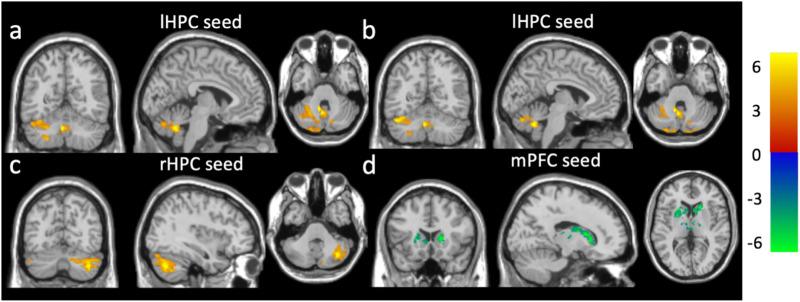
Association of rsFC with outcome variables at baseline (n = 18). Positive associations were observed (a) between lHPC-mlCB rsFC with NW speeds, (b) between lHPC-mlCB rsFC with DTW speeds, and (c) between rHPC-dlCB rsFC with HbA1c. A negative association was observed (d) between mPFC-BG rsFC with HOMA-IR. The color bar shows the range of t values. lHPC: left hippocampus; rHPC: right hippocampus; mlCB: midline cerebellum; dlCB: dorsal lateral cerebellum; mPFC: medio-prefrontal cortex; BG: basal ganglia; NW: normal walking; DTW: dual-task walking.

**Fig 6:**
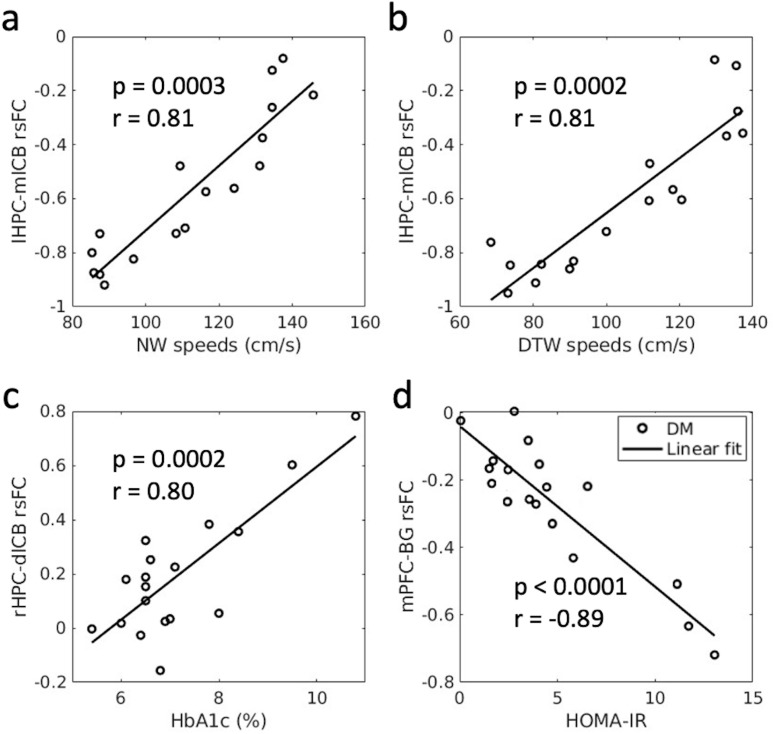
Post-hoc regional analyses (n = 18) showed significant associations between rsFC values and outcome variables at baseline. Positive associations were observed between (a) lHPC-mlCB rsFC and NW speeds, (b) lHPC-mlCB rsFC and DTW speeds, (c) between rHPC-dlCB rsFC with HbA1c. A negative association was observed (d) between mPFC-BG rsFC with HOMA-IR. lHPC: left hippocampus; rHPC: right hippocampus; mlCB: midline cerebellum; dlCB: dorsal lateral cerebellum; mPFC: medio-prefrontal cortex; BG: basal ganglia; NW: normal walking; DTW: dual-task walking.

In all 18 T2DM participants at baseline, we observed positive associations between lHPC-mlCB rsFC and NW speeds (Fig 5a, p = 0.0374), between lHPC-mlCB rsFC and DTW speeds (Fig 5b, p = 0.0192), and between rHPC-dlCB rsFC and HbA1c (Fig 5c, p = 0.0452, indicating larger rsFC with a worsening of diabetes control). A negative association was observed between mPFC-BG (basal ganglia) rsFC with HOMA-IR (Fig 5d, p = 0.049), which suggests that larger rsFC values between these regions are associated with lower levels of HOMA-IR, a measure of insulin resistance. The post-hoc regional analyses indicated that there exist statistically significant associations between the baseline rsFC values and these baseline variables (Fig 6a-d). Baseline mPFC-BG rsFC showed only weak associations with sex (p = 0.044) and antihypertensive drugs (p = 0.048) (see S3 Table for detailed p-values and partial Pearson correlation coefficients). After adjusting each covariate, the baseline rsFC values and outcome variables remain significant.

### Association of rsFC changes with diabetes variable changes from the baseline to end-of-treatment

**Fig 7:**
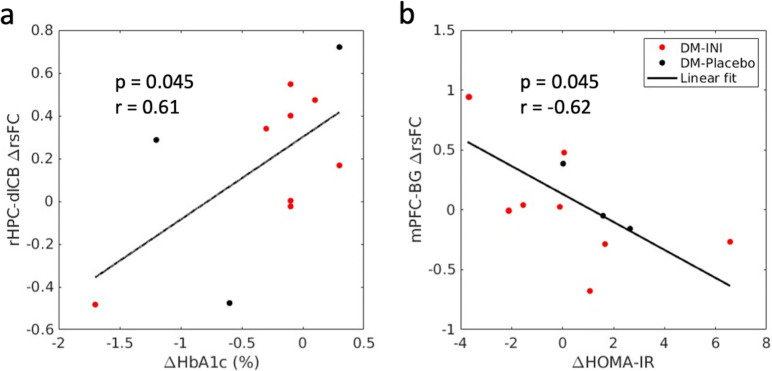
Regional analyses (n = 11) showed (a) positive association between changes in the rHPC-dlCB rsFC and HbA1c changes and (b) negative association between changes in the mPFC-BG rsFC and HOMA-IR changes. The black line shows the linear fitting for DM-INI and DM-Placebo subjects. rHPC: right hippocampus; dlCB: dorsal lateral cerebellum. mPFC: medio-prefrontal cortex; BG: basal ganglia.

Significant associations were found between changes in rHPC-dlCB rsFC and HbA1c changes (Fig 7a, r = 0.61, p = 0.045) and between changes in mPFC-BG rsFC and HOMA-IR changes (Fig 7b, r = -0.62, p = 0.045). Changes in rHPC-dlCB rsFC showed a weak association with antidepressant use (p = 0.047), while changes in mPFC-BG rsFC were associated with injectable antidiabetic drugs (p = 0.021) and lipid-lowering medications (p = 0.025) (see S4 Table for detailed p-values and partial Pearson correlation coefficients). After adjusting for injectable antidiabetic drugs, the association between changes in mPFC-BG rsFC and HOMA-IR changes becomes insignificant (p = 0.48). However, all other associations remain significant after adjusting for each covariate.

## Discussion

This study investigated the rsFC changes of T2DM patients treated with intranasal insulin. It explored the relationships between baseline rsFC and factors such as cognition, walking speeds, HbA1c, and HOMA-IR. It also examined the connections between the longitudinal changes in these factors after treatment and their respective rsFC alternations using resting state fMRI. Compared to the placebo treatment, INI treatment increases mPFC-postcentral rsFC, lHPC-frontal rsFC, lHPC-postcentral rsFC, rHPC-frontal rsFC, as well as increases lHPC-mPFC rsFC.

Previous studies reported reduced mPFC rsFC [39] and PCC rsFC [39,40] with precentral gyrus in T2DM, as compared to the controls. Due to the lack of a control group, this reduced rsFC in T2DM could not be verified in our fMRI sub-study. We found that INI increased mPFC-postcentral/precentral rsFC in patients with T2DM. The postcentral gyrus, a key component of the sensorimotor network, has been linked to gait speed in previous research, both in terms of gray matter density [41] and its connectivity with the mPFC [42], underscoring its critical role in sensory processing and motor function. In our study, we observed increased gait speed within the MEMAID cohort, potentially associated with enhanced rsFC between the mPFC and postcentral regions. Moreover, T2DM often leads to diabetic sensory neuropathy (DSN), a complication marked by impaired sensory and motor functions closely tied to insulin resistance [43]. By modulating rsFC between the mPFC and postcentral areas, intranasal insulin may enhance motor and sensory function in individuals with DSN, presenting a promising avenue for future research into the neural mechanisms underlying DSN. Obesity has been also associated with altered mPFC-postcentral rsFC [44], indicating heightened reactivity to food stimuli in overweight participants [45]. We verified that the increases in the mPFC-postcentral rsFC induced by INI were associated with the decreases in BMI (not shown). However, we did not observe the decreases in BMI in the MemAID study and thereby the effect of INI on mPFC-postcentral rsFC needs to be verified in large studies.

We found that 24-week INI treatment increased lHPC-mPFC rsFC, lHPC-frontal rsFC, and rHPC-frontal rsFC. This result extends the acute effect of a single-dose INI treatment in our prior study [24] to the long-term effect of INI treatment. Stronger rHPC-frontal rsFC after single-dose INI treatment was found to be associated with better verbal fluency. Moreover, T2DM patients showed decreased functional connectivity in their working memory networks, including the middle/superior frontal region, and the activation strength was significantly correlated with memory performance [46]. The frontal region is a key component of the default mode network (DMN), which plays a crucial role in self-referential thinking, memory processing, and cognitive flexibility. Research has shown that individuals with T2DM often exhibit disrupted rsFC within the DMN [47,48], which has been linked to impairments in memory and mental flexibility [49]. The MEMAID trial demonstrated the long-term benefits of INI on verbal memory [26], suggesting that INI may enhance DMN functional connectivity, thereby contributing to improvement in verbal memory. However, no significant association was observed between HPC-frontal rsFC and verbal memory scores in this study. The lack of association with verbal memory scores may be caused by the reduced sample size and is worth further investigation.

In T2DM participants, faster walking speed correlates with larger lHPC-mlCB rsFC at baseline. T2DM has been associated with reduced rsFC between mPFC and cerebellum [12,39], and between PCC and cerebellum [39,50]. The association of midline cerebellum rsFC with walking speed is consistent with the role of midline cerebellum on gait control and body movement [51,52]. Our results also support the involvement of HPC in gait performance, corroborating the cognitive recruitment in the hippocampus to coordinate motor performance and mobility [53,54]. Together with the association of HPC-midline cerebellum rsFC with walking speeds (NW and DTW speeds) at baseline, the increased trend of HPC-midline cerebellum rsFC after INI treatment observed in our sub-study (p = 0.13) indicates that the HPC-midline cerebellum rsFC could be a potential indicator for the effect of INI treatment on gait performance. Our MemAID trial has shown that INI treatment was associated with faster NW and DTW speeds in T2DM participants (n = 57) and with faster NW in non-diabetic controls (n = 65) [26]. In our small sample, however, the changes in walking speed were not significant. Therefore, this neuroimaging study may provide insights into the mechanisms underlying faster gait speeds after INI treatment in T2DM patients, specifically through improved HPC-midline cerebellum rsFC.

Lower HOMA-IR correlates with larger mPFC-BG rsFC in T2DM participants at baseline. In addition, the longitudinal increases in the mPFC-BG rsFC were associated with decreases in HOMA-IR over time. HOMA-IR has been reported to acutely modulate the effect of Intranasal insulin on midbrain circuitry (midbrain-mPFC rsFC) [55]. Considering that insulin receptors are abundant in the dopaminergic neurons of the BG/midbrain [56], our results corroborate that the altered HOMA-IR can impact on dopaminergic projections of the midbrain. Dopaminergic dysfunction in insulin-resistant states is linked to reduced motivation and impaired decision-making [57]. Collectively, these results suggest that insulin sensitivity and its affected midbrain circuitry may underlie disruptions in reward-related and motivational behavior in individuals with T2DM [58,59]. In the MemAID trial, HOMA-IR declined in the T2DM group after INI treatment [26], indicating the potential long-term effect of INI treatment on improving midbrain circuitry and ensuing motivational drive to reduce food intake [60].

We also found that longitudinal increases in the rHPC-dorsal lateral cerebellum rsFC were associated with longitudinal HbA1c increases, suggesting that hyperglycemia contributes to brain rsFC abnormalities. Considering that both HPC and mPFC are key regions of the default mode network, this finding is supported by a previous study [12] reporting that mPFC-dorsal lateral cerebellum rsFC had a significant negative correlation with HbA1c in T2DM with cognitive impairment despite opposite polarity. However, we did not find changes in HPC-dorsal lateral cerebellum rsFC after INI treatment, so the potential role of INI on cognitive function and hyperglycemia control is worth further investigation. The lack of observing these rsFC changes after INI treatment could be caused by our reduced sample size with INI treatment and therefore largely reduced statistical power.

**Fig 8:**
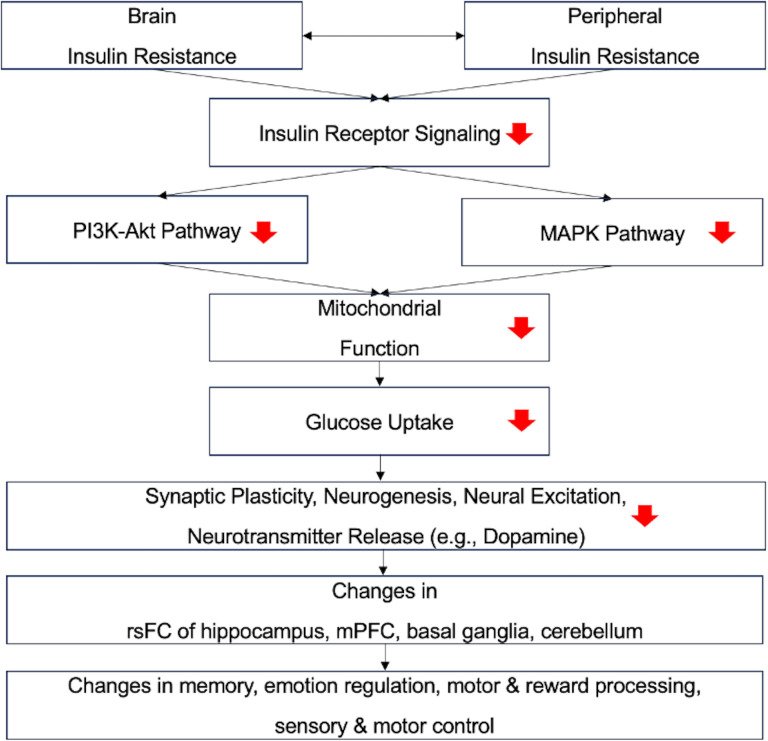
A conceptual model illustrating how insulin resistance, commonly associated with type 2 diabetes, affects brain connectivity and contributes to cognitive decline. Brain and peripheral insulin resistance interact, leading to reduced receptor signaling and disruption of critical cellular pathways. Downregulation of the PI3K-Akt pathway [66] (crucial for cell survival and metabolism [65]) and the MAPK pathway [63] (important for cell growth and differentiation [62]) impairs mitochondrial function [64], reducing ATP production. The energy deficit hinders glucose uptake [61], negatively impacting synaptic plasticity, neurogenesis, neural excitation, and neurotransmitter release (such as dopamine). We propose that brain regions rich in insulin receptors—such as the medial prefrontal cortex, hippocampus, basal ganglia, and cerebellum—are particularly vulnerable, as their glucose transport may largely depend on insulin-mediated pathways. These neuronal changes disrupt brain information transmission (functional connectivity), ultimately leading to impairments in memory, emotion regulation, motor control, reward processing, and sensory function.

Based on molecular pathways [61–66], we propose a conceptual model (Fig 8) illustrating how insulin resistance, commonly associated with type 2 diabetes, affects brain connectivity and contributes to cognitive decline. Intranasal insulin can modulate brain activity by bypassing the blood-brain barrier through direct transport along the olfactory and trigeminal nerve pathways. Once in the brain, insulin can bind to its receptors, which are abundant in key regions such as the medial prefrontal cortex, hippocampus, basal ganglia, and cerebellum. By influencing cellular processes like neurogenesis, synaptic plasticity, neural firing, and neurotransmitter release, intranasal insulin facilitates neural communication between these areas. This mechanism may enhance cognitive function by improving information processing and neural signaling across these brain regions. In the MEMAID trial, long-term intranasal insulin treatment reduced plasma insulin levels, suggesting potential systematic effects on brain activity through its regulation of peripheral insulin.

This study has limitations. Enrollment in the MRI sub-study was optional, allowing participants to decline, which may have introduced motivation bias. Limited funding prevented MRI scans for all participants, restricting the sub-study to a smaller group. However, demographic analysis revealed no statistically significant differences between those included in the MRI sub-study and the MEMAID clinical trial population. The sample size of INI and Placebo treated participants is limited, due to terminated MRI scans in March of 2020 due to the COVID-19 pandemic. To mitigate the potential biases arising from this attrition, we employed nonparametric statistical methods in our analysis. Nonparametric tests are more robust to the impact of missing data and are less sensitive to outliers or assumptions of normality, which helps provide more reliable results in the presence of attrition. Additionally, we carefully examined the characteristics of participants who were lost to follow-up and found no significant differences in baseline characteristics between those who completed the study and those who did not, which further supports the robustness of our findings. Our prior study identified a correlation between specific cognitive fields—verbal fluency and visuospatial memory—and rsFC between HPC and parts of DMN following a single dose of INI/placebo on T2DM patients. Therefore, we anticipate that verbal memory will be associated with DMN FC in T2DM at baseline. However, we did not observe this association in this current study. The small sample size may limit our ability to detect the association between verbal memory and HPC-DMN rsFC in T2DM, increasing the risk of Type II errors (false negatives) and Type I errors (false positives). Nevertheless, we applied the permutation tests (SnPM) to enhance the precision of our analyses. Unfortunately, we lacked a control group due to a budget cut. Without a control group, it is not possible for us to determine which regions exhibited compromised HPC/mPFC rsFC due to T2DM and correlate them with verbal memory. Consequently, the voxel-based analysis was adopted to identify rsFC deficits related to cognition; however, this approach suffers from a low signal-to-noise ratio, resulting in decreased sensitivity. Three DM-INI patients experienced worse glycemic control, unrelated to INI, as reflected in the insignificant differences between DM-INI and DM-Placebo for changes in plasma insulin, HOMA-IR, fasting glucose, normal walking, and dual-task walking. Therefore, we cannot rule out the effect of INI on rsFC between these seed regions and many other regions and the association of INI-induced rsFC changes and changes in cognition and gait speeds. Larger sufficiently powered studies need to delineate the effects of INI on rsFC in both older adults with T2DM and healthy older adults and to relate these to clinically significant outcomes. Additionally, exploring associations between brain MRI features and cognitive function using univariate correlation analyses faces significant limitations, particularly when dealing with small sample sizes [67]. In contrast, studies utilizing multivariate approaches, such as sparse canonical correlation analysis and partial least squares, have demonstrated greater effect sizes for the associations between brain features and clinical ratings or cognitive functions across various psychiatric conditions [67–69]. These techniques can be particularly advantageous in smaller samples, provided appropriate cross-validation or replication methods are included. Future research should explore the application of these multivariate techniques to capture the complex relationships between brain networks and cognitive variables in individuals with T2DM, as well as to investigate the effect of INI on those associations.

## Conclusion

This MemAID trial placebo-controlled sub-study in older adults with T2DM has shown that long-term administration of INI increased regional resting-state functional connectivity with mPFC and HPC regions and their relationship with clinical outcomes. This study provided insights into how intranasal insulin influences function connectivity over the long term in older diabetic adults, potentially underlying its cognitive and metabolic benefits.

## Supporting information

S1 Table Comparisons of demographic characteristics of the diabetes groups. (DOCX)

S2 Table Associations between covariates and changes in rsFC from baseline to the end of treatment in T2DM subjects (n = 11). (DOCX)

S3 Table Associations between covariates and baseline rsFC in T2DM subjects (n = 18). (DOCX)

S4 Table Associations between covariates and changes in rsFC in T2DM subjects (n = 11). (DOCX)

## Abbreviation:

ALFF: Amplitude of low-frequency fluctuations
bPC: Bilateral postcentral
BG: Basal ganglia
BMI: Body mass index
BOLD: Blood oxygen level-dependent
BRAVO: 3D T1-weighted brain volume
dlCB: Dorsal lateral cerebellum
DTW: Dual-task walking
FFT: Fast fourier transform
HbA1c: Hemoglobin A1c
HOMA-IR: Homeostatic model assessment of insulin resistance
HPC: Hippocampus
INI: Intranasal insulin
lHPC: Left hippocampus
lPOC: Left postcentral
lPRC: Left precentral
mlCB: Midline cerebellum
mPFC: Medio-prefrontal
MNI: Montreal neurological institute
MRI: Magnetic resonance imaging
NW: Normal walking
PCASL: Pseudo-continuous arterial spin labeling
rHPC: Right hippocampus
rPOC: Right postcentral
rPRC: Right precentral
rsFC: Resting-state functional connectivity
ROI: Region of interest
SnPM: Statistical nonparametric mapping
T2DM: Type 2 diabetes mellitus
