# Supplementary material for: Intranasal insulin enhances resting-state functional connectivity in Type 2 Diabetes
Source: PLoS One. 2025 May 20;20(5):e0324029. doi: 10.1371/journal.pone.0324029 (PMC12091882; doi:10.1371/journal.pone.0324029)
Supplement: S1 Table — (DOCX) [file pone.0324029.s001.docx]

**S1 Table.** Comparisons of demographic characteristics of the diabetes groups.

| Baseline data  DM participants | Included in  the sub-study  (n=18) | Excluded in  the sub-study  (n=88) | P value of  incl. vs. excl.  (n=18) vs. (n=88) | With follow-up  (n=11) | Without follow-up  (n=7) | P value of with vs. without follow-up  (n=11) vs. (n=7) |
| --- | --- | --- | --- | --- | --- | --- |
| Age(years) | 64.06±6.97 | 64.82±8.90 | 0.73 | 64.64±5.75 | 63.14±8.99 | 0.67 |
| Male/Female | 10/8 | 47/41 | 0.86 | 7/4 | 3/4 | 0.42 |
| Hypertension  /Normotension | 12/6 | 59/29 | 0.97 | 7/4 | 5/2 | 0.75 |
| ­Diabetes years | 10.44±6.71 | 11.01±7.68 | 0.77 | 9.18±5.27 | 12.43±8.28 | 0.33 |
| BMI (kg/m^2^) | 30.25±4.23 | 32.4±6.7 | 0.19 | 31.05±4.00 | 29.00±4.60 | 0.33 |
| Insulin (uIU/ml) | 11.89±8.51 | 18.8±24.52 | 0.24 | 10.07±5.85 | 14.76±11.50 | 0.27 |
| Fasting Glucose (mg/dl) | 149.61±37.68 | 141±46.21 | 0.48 | 152.18±39.92 | 145.57±36.53 | 0.73 |
| HOMA-IR | 4.71±3.69 | 6.62±8.07 | 0.33 | 4.02±2.86 | 5.80±4.77 | 0.34 |
| HbA1c (%) | 7.16±1.32 | 7.2±1.4 | 0.89 | 7.08±1.44 | 7.27±1.23 | 0.78 |
| NW speeds (cm/s) | 112.72±21.09 (n’=17) | 106.6±22.51 | 0.30 | 114.52±18.13  (n’=10) | 110.16±26.07 | 0.69 |
| DTW speeds (cm/s) | 105.45±24.59 (n’=17) | 97.1±23.23 | 0.18 | 107.22±20.63  (n’=10) | 102.93±31.00 | 0.74 |
| Verbal memory z score | -1.15±2.77 | -0.75±2.90 | 0.58 | -2.17±2.68 | 0.76±1.51 | **0.018** |
| Executive function z score | 0.59±2.23 | 1.25±2.20 | 0.25 | 0.54±2.15 | 0.48±2.77 | 0.96 |
