# Supplementary material for: Intranasal insulin enhances resting-state functional connectivity in Type 2 Diabetes
Source: PLoS One. 2025 May 20;20(5):e0324029. doi: 10.1371/journal.pone.0324029 (PMC12091882; doi:10.1371/journal.pone.0324029)
Supplement: S2 Table — (DOCX) [file pone.0324029.s002.docx]

**S2 Table.** Associations between covariates and changes in rsFC from baseline to the end of treatment in T2DM subjects (n = 11)

| P-values (r value)* | mPFC-lPOC rsFC | lHPC-frontal rsFC | lHPC-lPOC rsFC | rHPC-frontal rsFC | lHPC-mPFC rsFC |
| --- | --- | --- | --- | --- | --- |
| Age | 0.29 (0.40) | 0.16 (-0.51) | 0.099 (-0.58) | 0.15 (-0.52) | 0.80 (0.097) |
| Sex | 0.13 (-0.55) | 0.67 (0.16) | 0.99 (-0.0036) | 0.93 (0.034) | 0.79 (0.10) |
| BMI | 0.51 (-0.25) | 0.44 (0.30) | 0.55 (-0.23) | 0.29 (0.39) | 0.94 (-0.029) |
| Diabetes duration | 0.099 (-0.58) | 0.62 (-0.19) | 0.57 (0.22) | 0.19 (-0.48) | 0.31 (-0.38) |
| Hypertension | 0.57 (0.22) | 0.70 (-0.15) | 0.68 (-0.16) | 0.78 (-0.11) | 0.99 (0.0028) |
| Waist circumference | 0.32 (0.37) | 0.35 (0.35) | 0.65 (0.17) | 0.23 (0.45) | 0.42 (0.31) |
| Oral antidiabetic drugs | 0.45 (0.29) | 0.44 (-0.29) | 0.47 (-0.27) | 0.73 (0.13) | 0.80 (0.097) |
| Injectable antidiabetic drugs | 0.97 (-0.011) | 0.67 (-0.17) | 0.086 (0.60) | 0.49 (0.27) | 0.14 (-0.53) |
| Antihypertensive drugs | 0.29 (0.40) | 0.57 (-0.22) | 0.71 (-0.15) | 0.50 (-0.26) | 0.86 (-0.068) |
| Lipid lowering drugs | 0.081 (0.61) | 0.25 (-0.43) | 0.49 (-0.26) | 0.50 (-0.26) | 0.84 (0.077) |
| Antidepressants | 0.97 (0.015) | 0.40 (0.32) | 0.60 (-0.20) | 0.30 (0.39) | 0.55 (0.23) |

*The p-value is presented first, followed by the r-value in brackets.
