# Supplementary material for: Intranasal insulin enhances resting-state functional connectivity in Type 2 Diabetes
Source: PLoS One. 2025 May 20;20(5):e0324029. doi: 10.1371/journal.pone.0324029 (PMC12091882; doi:10.1371/journal.pone.0324029)
Supplement: S3 Table — (DOCX) [file pone.0324029.s003.docx]

**S3 Table.** Associations between covariates and baseline rsFC in T2DM subjects (n = 18)

| P-values (r value)* | lHPC-mlCB rsFC vs. NW speed | lHPC-mlCB rsFC vs. DTW speed | rHPC-dlCB rsFC  vs. HbA1c | mPFC-BG rsFC  vs. HOMA-IR |
| --- | --- | --- | --- | --- |
| Age | 0.15 (0.38) | 0.14 (0.38) | 0.34 (-0.25) | 0.16 (0.36) |
| Sex | 0.76 (-0.083) | 0.79 (-0.071) | 0.68 (-0.11) | **0.044 (0.49)** |
| BMI | 0.50 (0.18) | 0.47 (0.19) | 0.69 (-0.10) | 0.30 (0.26) |
| Diabetes duration | 0.14 (0.38) | 0.22 (0.33) | 0.83 (0.06) | 0.89 (-0.037) |
| Hypertension | 0.17 (-0.36) | 0.13 (-0.39) | 0.93 (0.024) | 0.074 (-0.44) |
| Waist circumference | 0.72 (0.096) | 0.60 (0.14) | 0.43 (-0.20) | 0.84 (0.052) |
| Oral antidiabetic drugs | NaN | NaN | 0.86 (-0.046) | 0.31 (0.26) |
| Injectable antidiabetic drugs | 0.38 (0.23) | 0.31 (0.27) | 0.97 (0.0089) | 0.45 (0.19) |
| Antihypertensive drugs | 0.82 (-0.062) | 0.85 (-0.050) | 0.34 (0.24) | **0.048 (-0.49)** |
| Lipid lowering drugs | 0.14 (0.39) | 0.17 (0.35) | 0.22 (0.32) | 0.72 (0.093) |
| Antidepressants | 0.73 (-0.092) | 0.76 (-0.084) | 0.69 (0.10) | 0.99 (0.0014) |

*The p-value is presented first, followed by the r-value in brackets. ^+^All subjects with NW speed or DTW speed acquired were on oral antidiabetic drugs. The bold fonts highlight the significant associations.
