# Supplementary material for: Intranasal insulin enhances resting-state functional connectivity in Type 2 Diabetes
Source: PLoS One. 2025 May 20;20(5):e0324029. doi: 10.1371/journal.pone.0324029 (PMC12091882; doi:10.1371/journal.pone.0324029)
Supplement: S4 Table — (DOCX) [file pone.0324029.s004.docx]

**S4 Table.** Associations between covariates and changes in rsFC in T2DM subjects (n = 11)

| P-values (r value)* | rHPC-dlCB $\Delta$rsFC vs. $\Delta$HbA1c | mPFC-BG $\Delta$rsFC  vs. $\Delta$HOMA-IR |
| --- | --- | --- |
| Age | 0.88 (0.057) | 0.87 (0.058) |
| Sex | 0.88 (0.055) | 0.40 (-0.29) |
| BMI | 0.57 (0.21) | 0.82 (0.082) |
| Diabetes duration | 0.86 (0.061) | 0.34 (-0.34) |
| Hypertension | 0.86 (0.063) | 0.22 (-0.42) |
| Waist circumference | 0.87 (0.059) | 0.94 (-0.029) |
| Oral antidiabetic drugs | 0.52 (0.23) | 0.16 (0.47) |
| Injectable antidiabetic drugs | 0.43 (-0.28) | **0.021 (0.71)** |
| Antihypertensive drugs | 0.83 (-0.078) | 0.46 (0.26) |
| Lipid lowering drugs | 0.62 (0.18) | **0.025 (0.69)** |
| Antidepressants | **0.047(-0.64)** | 0.095 (-0.55) |

*The p-value is presented first, followed by the r-value in brackets. The bold fonts highlight the significant associations.
